# A histone H2A docking domain mutant interferes with proper yFACT-gene interactions in *Saccharomyces cerevisiae*

**DOI:** 10.17912/micropub.biology.002322

**Published:** 2026-08-25

**Authors:** Lauren Joseph, Sydney A. Ozersky, McKenzie G. Tucker, Grace A. Turner, Michaela J. Edwards, Michelle L. Huynh, Andrea A. Duina

**Affiliations:** 1 Biology Department, Hendrix College, Conway, AR, United States

## Abstract

Alterations within the nucleosomal Influences Spt16-Gene Interactions (ISGI) region, which is located on the side of the nucleosome and is comprised of histone H3 and H4 residues, shift yFACT occupancy toward the 3′ ends of genes, likely due to defective yFACT dissociation following transcription. Here, we show that a single amino acid substitution within the histone H2A docking domain, H2A-I103A, similarly alters yFACT-gene interactions. These results demonstrate that histone H2A integrity is required for proper yFACT-gene interactions *in vivo* and suggest that the H2A docking domain promotes yFACT dissociation from genes.

**Figure 1. Overview of the genetic screen used in this work and its outcome, Spt16 occupancy across PMA1 and FBA1 in the context of different histone H2A and H2B mutants, and location of H2A-I103:** **(A)** Overview of the genetic screen used to identify H2A and H2B mutants that might interfere with proper yFACT-gene interactions. See text for details. **(B)** Location of the nine H2A and nine H2B residues across the corresponding proteins that when mutated to alanine cause growth defects in combination with the H3-L61T ISGI mutant. Alpha-helical regions (White et al. 2001) are indicated by the rectangles. **(C)** ChIP/qPCR assay to assess Spt16 occupancy across *PMA1* in the context of different histone H2A and H2B mutants. The cartoon on top depicts the *PMA1* locus, with “1” corresponding to the start of the gene’s coding region. The blue and orange bars show the two genomic locations that were assayed for Spt16 occupancy, and arrows indicate the direction of transcription. The bar-graphs on the left reflect Spt16 occupancy levels measured using ChIP/qPCR assays in cells expressing either wild-type or the indicated histone mutant – in each case, the occupancy level at the 5’ location (blue bars) was set to 1, and the occupancy level at the 3’ region (orange bars) was calculated relative to the corresponding 5’ occupancy level. The bar-graphs on the right are set up the same way as the left bar-graphs, but reflect the mean ± S.E.M. from three independent experiments carried out in wild-type and H2A-I103A strains, with the asterisk indicating a statistically significant difference (Student’s *t* -tests, *P* < 0.05). **(D)** Results from ChIP/qPCR experiments showing the effects of the H2A-I103A mutant on Spt16 occupancy across the *FBA1* gene. The data are displayed as described for the right bar-graph in panel A. **(E)** Side-view of the yeast nucleosome core particle, with the ISGI region shown in blue and one of the two H2A-I103 residues shown in green.

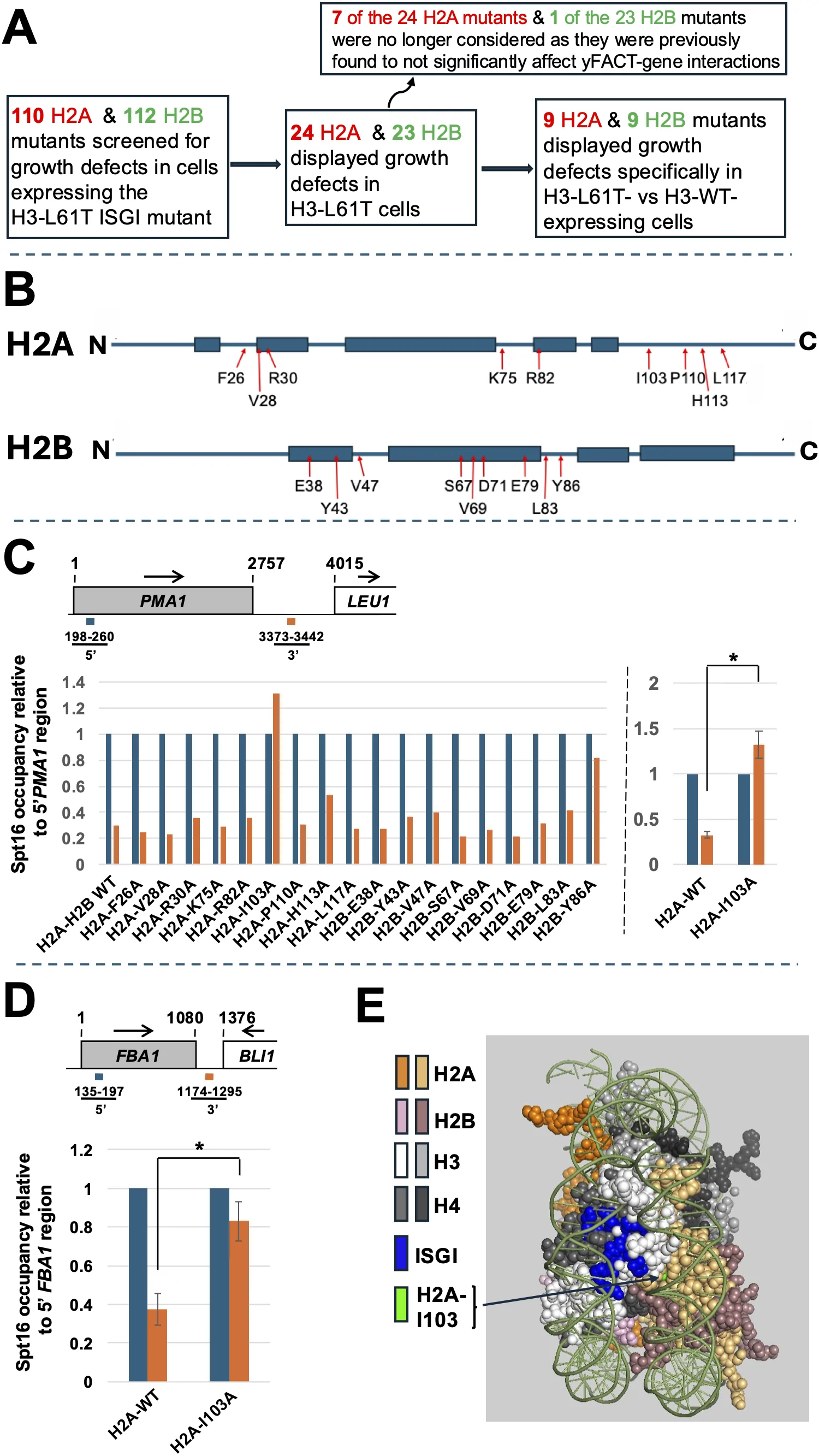



## Description

FACT (FAcilitates Chromatin Transcription/Transactions) is a histone chaperone complex that interacts with nucleosomes and coordinates a variety of chromatin-templated processes, including gene transcription, DNA replication, and DNA repair (recently reviewed in (Formosa and Winston 2020; Zhou et al. 2020; Wang et al. 2021; Jeronimo and Robert 2022; Volokh et al. 2025)). During transcription, FACT has roles in facilitating both the disassembly of nucleosomes ahead of RNA polymerase II (Pol II) and in their reassembly in the wake of Pol II passage, the latter process being carried out using the original histones, thus ensuring maintenance of nucleosomal epigenetic codes. Whereas many elegant studies have provided a wealth of information about FACT/nucleosome interactions *in vitro* (for some recent insights into this process, see (Liu et al. 2020; Farnung et al. 2021; Ehara et al. 2022; McCauley et al. 2022; Sivkina et al. 2022; Engeholm et al. 2024; Burgos-Bravo et al. 2025)), less is known regarding the details of how FACT interacts with nucleosomes and chromatin in an *in vivo* setting. In our previous work using the budding yeast model system, we have identified a region on the side of the nucleosome whose integrity is required to promote proper yeast FACT (yFACT) interactions across transcribed genes. This region, which we named ISGI (Influences Spt16-Gene Interactions – note that Spt16 is part of yFACT), is composed of histone H3 and H4 residues, and alterations within it cause a shift in yFACT distribution towards the 3’ ends of genes, a defect we have attributed to impairment in yFACT dissociation from genes following transcription (Nguyen et al. 2013; Nyamugenda et al. 2018).

To assess whether other nucleosomal regions, in particular those composed of histone H2A and H2B residues, also impact yFACT-gene interactions, we carried out a genetic screen, summarized in Figure 1A, designed to identify H2A and H2B mutants that impair yFACT interaction across transcribed genes in a manner similar to that seen in ISGI mutants. For these experiments, we transformed a strain harboring a deletion of both loci encoding histones H2A and H2B ( *i.e., HTA1-HTB1* and *HTA2-HTB2)* and carrying a *URA3-* marked plasmid containing the wild-type *HTA1-HTB1* genes with a previously described *HIS3-* marked plasmid library expressing histone H2A or H2B mutants (SHIMA library, (Nakanishi et al. 2008)). The host strain, called yADP165, also expresses the moderately strong ISGI mutant H3-L61T as its only source of histone H3 – while H3-L61T causes a moderate yFACT 3’-shift across genes, it does not confer the strong growth phenotypes that are observed among the stronger ISGI mutants, such as H3-L61W, H3-L61R, or H4-R36A (Duina et al. 2007; Nguyen et al. 2013; Johnson et al. 2015). For our screen, we thus reasoned that any H2A or H2B mutants that cause even a small degree of yFACT 3’-shift would, in combination with the H3-L61T mutant, cause a sufficiently strong yFACT 3’-shift that would result in growth phenotypes that we could easily detect. Histone H2A and H2B mutant candidates resulting from this screen could then be assayed directly for defects in yFACT-gene interactions.

Following the transformation of the SHIMA library into strain yADP165, transformants were transferred to 5-FOA-containing media to select for loss of the *URA3* plasmid harboring the *HTA1-HTB1* genes and assayed for growth phenotypes associated with the strongest ISGI mutants: viability, cold sensitivity, and sensitivity to the drugs caffeine, hydroxyurea, and formamide. From a total of 110 H2A mutants and 112 H2B mutants screened, 47 showed at least a subset of these growth phenotypes. Eight of these mutants had not shown significant defects in yFACT-gene interactions in previous experiments in our lab and were therefore no longer considered. The remaining 39 mutants were subjected to a secondary screen that was carried out the same way as the first screen but using a host strain expressing wild-type histone H3 instead of H3-L61T (strain yADP166) – mutants displaying more pronounced growth phenotypes in H3-L61T cells compared to wild-type H3 cells were further analyzed as these were more likely to confer growth defects through exacerbation of the H3-L61T-mediated yFACT 3’-shift across genes.

The surviving candidates, 9 H2A mutants and 9 H2B mutants (see locations of the corresponding residues in Figure 1B ), were then subjected to chromatin immunoprecipitation (ChIP) assays followed by qPCR analysis to assess occupancy levels of the yFACT subunit Spt16 across *PMA1* , a highly and constitutively transcribed gene commonly used to study transcription factor occupancies across genes. These initial experiments, which were done on a single sample per mutant, highlighted H2A-I103A as a mutant that causes a shift of yFACT occupancy toward the 3’ end of *PMA1* ( Figure 1C, bar-graph on the left side). To confirm this effect, we carried out Spt16 ChIP/qPCR on two additional independent H2A-I103A samples – as shown on the bar-graph on the right side of Figure 1C, the aggregate data for the three H2A-I103A samples show that this mutant causes a > 4-fold shift in yFACT occupancy towards the 3’ end of *PMA1* . To ensure that the effect of H2A-I103A on yFACT occupancy across *PMA1* is not unique to this gene but a more general phenomenon, we assayed its effects across *FBA1* , another gene used as a model for highly and constitutively transcribed genes. As shown in Figure 1D, the H2A-I103A mutant also caused a 3’-shift of yFACT across *FBA1* , an effect that was measured to be over 2-fold. Thus, the H2A-I103A mutant causes a 3’-shift in yFACT occupancy across at least two transcribed genes in a manner that is similar to that seen in the context of histone H3 and H4 ISGI mutants, suggesting that this histone mutant interferes with proper yFACT dissociation from genes following transcription. Interestingly, in the context of the screen, H2A-I103A conferred a synthetic lethal (or extremely sick) phenotype in combination with the H3-L61T ISGI mutant, possibly indicating that the combined mutants cause yFACT dissociation defects severe enough to cause lethality, a phenotype also displayed by one of the strongest ISGI mutant we have thus far identified, H3-L61R (Pablo-Kaiser et al. 2022).

The H2A-I103 residue resides within the H2A docking domain – a region located toward the carboxy terminus of the protein that forms an interacting surface with the H3-H4 tetramer within the nucleosome ( Figure 1E ) (Luger et al. 1997; Ehara et al. 2022) and that has been previously shown to genetically interact with yFACT (VanDemark et al. 2008). Recent structural studies have shown that as Pol II and associated transcription factors traverse a nucleosome, the middle domain of Spt16 (Spt16 MD) interacts with surfaces of the histone H3-H4 tetramer and an H2A-H2B dimer and in so doing disrupts interactions between the H2A docking domain and histone H3 (Ehara et al. 2022) – it is therefore possible that the H2A-I103A mutant could interfere with these dynamic processes in some manner, ultimately leading to the inability of yFACT to dissociate efficiently from DNA at the end of the transcription process. Since H2A-I103 is relatively near the ISGI region ( Figure 1E ), it is also possible that the H2A-I103A mutant exerts its effects on yFACT-gene interactions through perturbations of the ISGI region. Previous research has reported that the H2A-I103A mutant confers phenotypes indicative of defects in transcription (Spt ^-^ phenotype), DNA replication (hydroxyurea sensitivity), and DNA repair (methyl methanesulfonate sensitivity) (Sakamoto et al. 2009), and, given yFACT’s widespread role in chromatin processes, it is possible that some or all of these phenotypes are related to yFACT abnormal retention at 3’ ends of genes. Overall, our results indicate that histone H2A integrity, in particular within its docking domain, is required for proper yFACT-gene interactions in an *in vivo* setting.

## Methods

***Yeast strains, genetic methods, and media:*** All yeast strains used in this study are *GAL2 ^+^* derivatives of the S288C strain background (Winston et al. 1995) and their genotypes are presented in the Reagents section. We note that we also generated strains with the *hta2* allele expressing the H2A-I103A mutant integrated into the genome – however, because of unexpected segregation patterns of the mutant allele in subsequent genetic crosses (likely due to an event related to the known phenomenon of circular chromosome formation in cells deleted for the *HTA1-HTB1* locus (Libuda and Winston 2006)) the strains used in this work express H2A-I103A (as well as the other H2A and H2B mutants) from plasmids. Standard genetic techniques and media preparation protocols have been described previously (Rose et al. 1990).

***Chromatin Immunoprecipitation (ChIP)/qPCR assays:*** ChIP/qPCR assays to assess occupancy of Spt16 across the *PMA1* and *FBA1* genes were carried out on strains yADP166-yADP184 as previously described (Myers et al. 2011), except that chromatin was sheared to an average size of ~300-400 using a Bioruptor 300 (Diagenode) and that antibody-chromatin complexes were isolated using Protein G-coated dynabeads (Thermo Fisher Scientific, Catalog #10004D). The following primer sets were used for the qPCR analysis: *5’PMA1* , OAD394 and OAD395; *3’PMA1* , OAD383 and OAD384; *5’FBA1,* OAD419 and OAD420; *3’FBA1* , OAD423 and OAD424 – the sequences for these primers have been provided in previous work (Myers et al. 2011; Nguyen et al. 2013).

***Visualization of the nucleosome core particle and relevant residues:*** The structure shown in Figure 1E was generated using PyMOL Molecular Graphics System, Version 1.5.0.3 Schrödinger, LLC using previously published structural information ((White et al. 2001) and available at the Research Collaboratory for Structural Bio-informatics protein data bank (PDB ID:1id3).

## Reagents

***Antibodies used in ChIP assays:*** ChIP assay were carried out using polyclonal antibodies specific for the yeast Spt16 protein (a gift from Tim Formosa).

***Saccharomyces cerevisiae strains used in this study:***

**Table:** 

**Strain Name**	** Genotype**	** Source**
yADP165	*MAT* α; his3∆200; leu2∆1; ura3*; trp1∆63; lys2-128δ; (hta1-htb1)∆::LEU2; (hta2-htb2)∆::TRP1; (hht1-hhf1)∆::NatMX; hht2 (H3-L61T); p(HTA1-HTB1)-URA3	This study
yADP166	*MAT* α; his3∆200; leu2∆1; ura3*; trp1∆63; lys2-128δ; (hta1-htb1)∆::LEU2; (hta2-htb2)∆::TRP1; (hht1-hhf1)∆::NatMX; HHT2 (H3-WT); p(HTA1-HTB1)-URA3	This study
yADP167	*MAT* α; his3∆200; leu2∆1; ura3*; trp1∆63; lys2-128δ; (hta1-htb1)∆::LEU2; (hta2-htb2)∆::TRP1; (hht1-hhf1)∆::NatMX; HHT2 (H3-WT); p(hta1(H2A-F26A)-HTB1(H2B-WT))-HIS3	This study
yADP168	*MAT* α; his3∆200; leu2∆1; ura3*; trp1∆63; lys2-128δ; (hta1-htb1)∆::LEU2; (hta2-htb2)∆::TRP1; (hht1-hhf1)∆::NatMX; HHT2 (H3-WT); p(hta1(H2A-V28A)-HTB1(H2B-WT))-HIS3	This study
yADP169	*MAT* α; his3∆200; leu2∆1; ura3*; trp1∆63; lys2-128δ; (hta1-htb1)∆::LEU2; (hta2-htb2)∆::TRP1; (hht1-hhf1)∆::NatMX; HHT2 (H3-WT); p(hta1(H2A-R30A)-HTB1(H2B-WT))-HIS3	This study
yADP170	*MAT* α; his3∆200; leu2∆1; ura3*; trp1∆63; lys2-128δ; (hta1-htb1)∆::LEU2; (hta2-htb2)∆::TRP1; (hht1-hhf1)∆::NatMX; HHT2 (H3-WT); p(hta1(H2A-K75A)-HTB1(H2B-WT))-HIS3	This study
yADP171	*MAT* α; his3∆200; leu2∆1; ura3*; trp1∆63; lys2-128δ; (hta1-htb1)∆::LEU2; (hta2-htb2)∆::TRP1; (hht1-hhf1)∆::NatMX; HHT2 (H3-WT); p(hta1(H2A-R82A)-HTB1(H2B-WT))-HIS3	This study
yADP172	*MAT* α; his3∆200; leu2∆1; ura3*; trp1∆63; lys2-128δ; (hta1-htb1)∆::LEU2; (hta2-htb2)∆::TRP1; (hht1-hhf1)∆::NatMX; HHT2 (H3-WT); p(hta1(H2A-I103A)-HTB1(H2B-WT))-HIS3	This study
yADP173	*MAT* α; his3∆200; leu2∆1; ura3*; trp1∆63; lys2-128δ; (hta1-htb1)∆::LEU2; (hta2-htb2)∆::TRP1; (hht1-hhf1)∆::NatMX; HHT2 (H3-WT); p(hta1(H2A-P110A)-HTB1(H2B-WT))-HIS3	This study
yADP174	*MAT* α; his3∆200; leu2∆1; ura3*; trp1∆63; lys2-128δ; (hta1-htb1)∆::LEU2; (hta2-htb2)∆::TRP1; (hht1-hhf1)∆::NatMX; HHT2 (H3-WT); p(hta1(H2A-H113A)-HTB1(H2B-WT))-HIS3	This study
yADP175	*MAT* α; his3∆200; leu2∆1; ura3*; trp1∆63; lys2-128δ; (hta1-htb1)∆::LEU2; (hta2-htb2)∆::TRP1; (hht1-hhf1)∆::NatMX; HHT2 (H3-WT); p(hta1(H2A-L117A)-HTB1(H2B-WT))-HIS3	This study
yADP176	*MAT* α; his3∆200; leu2∆1; ura3*; trp1∆63; lys2-128δ; (hta1-htb1)∆::LEU2; (hta2-htb2)∆::TRP1; (hht1-hhf1)∆::NatMX; HHT2 (H3-WT); p(HTA1(H2A-WT)-htb1(H2B-E38A))-HIS3	This study
yADP177	*MAT* α; his3∆200; leu2∆1; ura3*; trp1∆63; lys2-128δ; (hta1-htb1)∆::LEU2; (hta2-htb2)∆::TRP1; (hht1-hhf1)∆::NatMX; HHT2 (H3-WT); p(HTA1(H2A-WT)-htb1(H2B-Y43A))-HIS3	This study
yADP178	*MAT* α; his3∆200; leu2∆1; ura3*; trp1∆63; lys2-128δ; (hta1-htb1)∆::LEU2; (hta2-htb2)∆::TRP1; (hht1-hhf1)∆::NatMX; HHT2 (H3-WT); p(HTA1(H2A-WT)-htb1(H2B-V47A))-HIS3	This study
yADP179	*MAT* α; his3∆200; leu2∆1; ura3*; trp1∆63; lys2-128δ; (hta1-htb1)∆::LEU2; (hta2-htb2)∆::TRP1; (hht1-hhf1)∆::NatMX; HHT2 (H3-WT); p(HTA1(H2A-WT)-htb1(H2B-S67A))-HIS3	This study
yADP180	*MAT* α; his3∆200; leu2∆1; ura3*; trp1∆63; lys2-128δ; (hta1-htb1)∆::LEU2; (hta2-htb2)∆::TRP1; (hht1-hhf1)∆::NatMX; HHT2 (H3-WT); p(HTA1(H2A-WT)-htb1(H2B-V69A))-HIS3	This study
yADP181	*MAT* α; his3∆200; leu2∆1; ura3*; trp1∆63; lys2-128δ; (hta1-htb1)∆::LEU2; (hta2-htb2)∆::TRP1; (hht1-hhf1)∆::NatMX; HHT2 (H3-WT); p(HTA1(H2A-WT)-htb1(H2B-D71A))-HIS3	This study
yADP182	*MAT* α; his3∆200; leu2∆1; ura3*; trp1∆63; lys2-128δ; (hta1-htb1)∆::LEU2; (hta2-htb2)∆::TRP1; (hht1-hhf1)∆::NatMX; HHT2 (H3-WT); p(HTA1(H2A-WT)-htb1(H2B-E79A))-HIS3	This study
yADP183	*MAT* α; his3∆200; leu2∆1; ura3*; trp1∆63; lys2-128δ; (hta1-htb1)∆::LEU2; (hta2-htb2)∆::TRP1; (hht1-hhf1)∆::NatMX; HHT2 (H3-WT); p(HTA1(H2A-WT)-htb1(H2B-L83A))-HIS3	This study
yADP184	*MAT* α; his3∆200; leu2∆1; ura3*; trp1∆63; lys2-128δ; (hta1-htb1)∆::LEU2; (hta2-htb2)∆::TRP1; (hht1-hhf1)∆::NatMX; HHT2 (H3-WT);p(HTA1(H2A-WT)-htb1(H2B-Y86A))-HIS3	This study
	*This allele is either *ura3-52* or *ura3∆0*	
